# A 13-Bit, 12-ps Resolution Vernier Time-to-Digital Converter Based on Dual Delay-Rings for SPAD Image Sensor

**DOI:** 10.3390/s21030743

**Published:** 2021-01-22

**Authors:** Zunkai Huang, Jinglin Huang, Li Tian, Ning Wang, Yongxin Zhu, Hui Wang, Songlin Feng

**Affiliations:** Shanghai Advanced Research Institute, Chinese Academy of Sciences, Shanghai 201210, China; huangzk@sari.ac.cn (Z.H.); huangjl@sari.ac.cn (J.H.); tianl@sari.ac.cn (L.T.); wangning@sari.ac.cn (N.W.); zhuyongxin@sari.ac.cn (Y.Z.); fengsl@sari.ac.cn (S.F.)

**Keywords:** three-dimensional (3D) image sensor, time-to-digital converter (TDC), pulse-width self-restricted (PWSR) delay element, fully symmetric arbiter, vernier

## Abstract

A three-dimensional (3D) image sensor based on Single-Photon Avalanche Diode (SPAD) requires a time-to-digital converter (TDC) with a wide dynamic range and fine resolution for precise depth calculation. In this paper, we propose a novel high-performance TDC for a SPAD image sensor. In our design, we first present a pulse-width self-restricted (PWSR) delay element that is capable of providing a steady delay to improve the time precision. Meanwhile, we employ the proposed PWSR delay element to construct a pair of 16-stages vernier delay-rings to effectively enlarge the dynamic range. Moreover, we propose a compact and fast arbiter using a fully symmetric topology to enhance the robustness of the TDC. To validate the performance of the proposed TDC, a prototype 13-bit TDC has been fabricated in the standard 0.18-µm complementary metal–oxide–semiconductor (CMOS) process. The core area is about 200 µm × 180 µm and the total power consumption is nearly 1.6 mW. The proposed TDC achieves a dynamic range of 92.1 ns and a time precision of 11.25 ps. The measured worst integral nonlinearity (INL) and differential nonlinearity (DNL) are respectively 0.65 least-significant-bit (LSB) and 0.38 LSB, and both of them are less than 1 LSB. The experimental results indicate that the proposed TDC is suitable for SPAD-based 3D imaging applications.

## 1. Introduction

Recently, three-dimensional (3D) imaging has emerged as a pivotal technique in a wide range of scientific and industrial processes, such as autonomous robots, scientific research, consumer electronics, aerospace, medical imaging, autonomous vehicles, etc. [1,2,3,4,5] Generally, there are three basic approaches currently being adopted in research into 3D imaging, namely, structured light [6], binocular stereo vision [7], and time-of-flight (ToF) [8]. Compared to other techniques, ToF-based 3D imaging holds lots of superiorities, including compact construction, wide detectable range, low cost, rapid measuring time, low power consumption, etc. Hence, it has attracted growing attention from both academia and industry [9].

ToF techniques extract the depth information, employing measuring the time interval between the emission of the light source and its return to the time-resolved photon detector, after being reflected by the surface of the object. Usually, the light source is made up of a pulsed or a continuously modulated light emitter. In general, ToF techniques can be divided into two main categories: respectively, the direct ToF (D-ToF) technique [10] and the indirect ToF (I-ToF) technique [11]. I-ToF techniques estimate the depth information by measuring the phase-shift between the received pulse/modulated light and the emitted light through the agency of a complicated photo-demodulator pixel array. Practically, sequential acquisition of three or more sub-frames is required to properly achieve the modulation or demodulation schemes as well as obtain the depth information. I-ToF tends to be particularly more suitable for short-range, high resolution 3D imaging [12]; while the D-ToF approach is based on the measurement of the time required by a photon to travel from the illuminator towards a target and back to the photodetector. Typically, the illuminator is an accurate picosecond-width pulsed light source that sends a periodic laser pulse to the target. The photodetector is formed by avalanche photodiodes with the Geiger-mode and high-performance readout electronics. It converts the reflected laser photons to electrical signals with temporal and spatial information for 3D imaging. Generally, the photodetector is a crucial element in a D-ToF system.

As mentioned above, the D-ToF technique directly calculates the distance by the elapsed time between the emitted light pulse and the detection of the reflected light. Commonly, high time-resolution can be easily obtained by avalanche detectors without any ambiguity. Interference of the uncorrelated light can be also effectively eliminated by implementing smart techniques at pixel level [13]. Thus, D-ToF can provide longer measurement ranges and improved robustness against artifacts. Furthermore, recent advances in Single-Photon Avalanche Diode (SPAD) detectors in complementary metal–oxide–semiconductor (CMOS) technology indicate that high-sensitivity, low-noise devices can be combined with dense logic in small areas, even in combination with advanced packaging technique [14]. SPAD-based D-ToF systems have been grabbing enormous interest from researchers, and they are also identified as one of the most critical building blocks in systems like aerospace, fully autonomous car driving, and so on.

**Figure 1:**
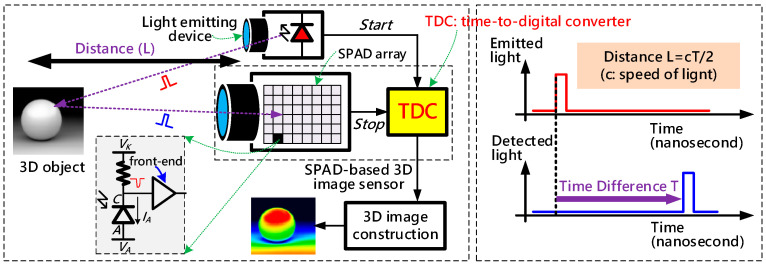
Structure and operation of a typical Single-Photon Avalanche Diode (SPAD)-based direct time-of-flight (D-ToF) system.

Figure 1 illustrates an example of a typical SPAD-based D-ToF system, which includes an object to be measured, a light-emitting device to illuminate the object, and a SPAD image sensor to collect the reflected light as well as evaluate the elapsed time. The distance between the object and the SPAD image sensor is determined by the elapsed time between emitted and received light and can be calculated by:

(1) L=cT/2,

where *L* represents the distance from the sensor to the measured object, *c* is the speed of light, and *T* is the time difference between the emitted and detected light pulse. As shown in Figure 1, the SPAD image sensor plays the most crucial role in constructing the D-ToF system. It is mainly composed of a SPAD array, a bank of time-to-digital converters (TDCs), a controller, and several output logics [15]. The SPAD array is responsible for providing true single-photon sensitivity and high frame rates, and it generates a fast pulse signal upon receiving the reflected light [16]. TDCs are principally utilized to measure the time-interval accurately [17]. Both SPAD array and TDCs are indispensable and essential for the SPAD image sensor, but, in this paper, we primarily focus on the design and optimization of TDC.

As its name suggests, TDC behaves like a digital stopwatch which precisely quantizes the time-interval between the ‘Start’ signal and ‘Stop’ signal into digital codes. As shown in Figure 1, the ‘Start’ signal is provided by the system clock as a reference of light-emitting, and the ‘Stop’ signal implies a photo hitting the sensor surface. For the TDC adopted in a SPAD image sensor, the design challenges include but are not limited to wide dynamic range, small die area, high time-resolution, and low power consumption. The TDC’s performance directly determined the imaging capability of the 3D image sensor. A high output range with fine resolution allows the sensor to capture realistic shapes and surface textures of objects [18]. For instance, if we need to detect an object with a single-shot distance resolution of 1 cm within up to 10 m away, a leading TDC whose time-resolution is smaller than 30 ps and dynamic range is wider than 10-bit is desired. This is a particularly tough requirement to meet.

TDC architectures can be mainly classified in terms of operation principle into three distinct types, which are: the digital method using fast counters [19], the analog method based on generating a voltage ramp [20], and the delay line method using high precision delay cell [17], respectively. Practically, the delay-line method is of particular interest because it presents superior characteristics over others, such as excellent salability, small cost, low power dissipation, and high compatibility with standard CMOS technologies. In recent years, a considerable amount of literature has been published on the delay-line-based TDCs. Specifically, a novel TDC architecture that reused vernier delay cells in a ring configuration for digital-phase-locked-loops was proposed in [21]. It achieved a large detectable range while keeping a high time precision. Additionally, a TDC architecture that was capable of reaching high-precision and high-linearity was developed in [22]. The proposed architecture innovatively employed a coarse counter and a single-stage fine interpolator to improve the conversion linearity. The measured results showed that time precision smaller than 17 ps was successfully achieved. Similarly, a folding-flash based on the remainder number system was proposed in [23]. The reported TCD employed a dual free-running oscillator to perform fine quantization. In this way, the hardware complexity was decreased significantly without speed impairment. Furthermore, a successive approximation TDC based on binary-scaled delay lines was reported in [24]. It effectively reduced the circuit complexity by tactfully removing one delay line in the feedforward architecture. Finally, the simulation results indicated that the reported TDC reached a nominal resolution of 25 ps and a dynamic range of 8-bit. Collectively, the TDCs presented in the state-of-the-art literature have indeed exhibited remarkable performances. Yet, there are still several issues requiring urgent solutions. Conventional delay-line-loop-based TDC usually suffers from low resolution owing to the restrictive clock frequency, and it may be suitable only for short dynamic range. Although the vernier technique overcomes the resolution limitations, it occupies a large die area and thus requires high power consumption. Employing a looped vernier structure can broaden the dynamic range, but extra stabilizing circuits and complex arbiter are required to compensate for the mismatches arising from process, voltage, and temperature (PVT) variations. This will inevitably increase the design effort and add non-linearity among other design issues.

In this paper, we design and implement a novel high-performance TDC for the SPAD Image Sensor. The main contributions of this work can be concluded as follows:

1. We propose a vernier TDC architecture based on dual delay-rings which can achieve a sub-gate resolution as well as an ultra-high dynamic range with a small area and low power consumption.
2. A pulse-width self-restricted (PWSR) delay element which is capable of providing accuracy and steady time delay is proposed to build the dual delay-rings.
3. We design a compact and fast arbiter that is tolerant of PVT variation using a fully symmetric topology for the proposed TDC.
4. We verify our design by taping-out a prototype TDC based on the proposed architecture and blocks using standard 0.18-µm CMOS technology.

The rest of this paper is organized as follows: Section 2 briefly introduces the overall architecture and operation principle of the proposed vernier type TDC for the SPAD image sensor. Section 3 thoroughly describes the circuit implementations of TDC’s critical blocks. In Section 4, the verification system is developed and the experimental results are discussed as well. Section 5 briefly introduces our subsequent work: a 3D image sensor based on the proposed TDC. Finally, conclusions are provided in Section 6.

## 2. Overall Architecture and Operation

**Figure 2:**
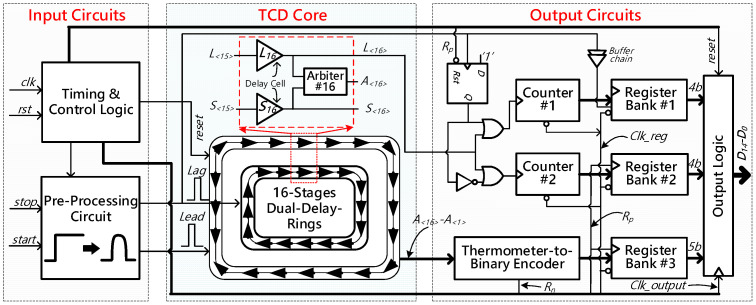
Principle block diagram of the proposed vernier time-to-digital converter (TDC).

Figure 2 principally illustrates the overall block diagram of our proposed vernier TDC. For ease of description, we manually divide the proposed TDC on the basis of their functions into three blocks, namely the input circuits, the TCD core, and the output circuits. The input circuits are mainly composed of the time and control logics and the pre-processing circuit. As its name implies, the time and control logics basically provide proper clock and control signals for other blocks in the TDC. Particularly, the pre-processing circuit is responsible for sharping the input signals (‘*Start*’ and ‘*Stop*’) to narrow pulses (‘*Lead*’ and ‘*Lag*’) whose widths are only hundreds of picoseconds. A quintessential pre-processing circuit is constructed by a D-type flip-flop (DFF) and some logic gates. In general, the original signals are fed into the TDC in the form of step waveforms or wide pulses. If they enter the delay-rings directly, the TDC might fail because the wide pulses will annihilate themselves in the delay-ring eventually after several rounds of propagation. In this case, the TDC’s dynamic range will be largely restricted. Hence, the pre-processing circuit is necessary for TDC.

As shown in Figure 2, the TDC core is regarded as the most critical block. It consists of a pair of delay-rings: one for the ‘*Lead*’ signal and the other for the ‘*Lag*’ signal. Here, the ‘*Lead*’ and ‘*Lag*’ signals inherit the temporal information of the input signals: ‘*Start*’ and ‘*Stop*’. The dual delay-rings are constructed with several basic modules, and each module is built up with two delay pairs and one pulse arbiter. Essentially, the delay elements in the delay-ring where the ‘*Lead*’ signal enters have a larger delay time than those in the delay-ring which receives the ‘*Lag*’ signal. The time skew measurement is triggered as the ‘*Lead*’ pulse appears and stopped once the ‘*Lag*’ signal catches up with the ‘*Lead*’ signal. The minimal measurable time-interval is mainly determined by both the delay elements and the pulse arbiter. Undoubtedly, the TDC core is a vital part of our proposed circuit, and its detailed architecture and principle will be fully discussed in Section 3.

The output circuits are depicted in the right part of Figure 2. They are chiefly composed of a thermometer-to-binary (TM2B) encoder, two sets of digital counters, several register banks, and output logic. Generally, the outputs of the arbiters in the TDC core are thermometer codes. The TM2B encoder can convert them to binary codes for ease of processing. For the two counters, the *1st* counter records the cycles when the ‘*Lead*’ signal propagates in the delay-ring alone, and the *2nd* one counts the cycles the ‘*Lead*’ signal and the ‘*Lag*’ signal propagate in the dual delay-rings together. Moreover, the three sets of register banks, as well as the output logic, are principally utilized for aligning, storing, and enhancing the digital outputs legitimately.

## 3. Circuit Implementation

This section will thoroughly describe the operating principles as well as the design strategies of the critical blocks in our proposed TDC. Specifically, the concerned blocks include the TDC core, the PWSR delay element, the full symmetric arbiter, and the compact TM2B encoder. Finally, this section will also provide some essential simulation results for preliminary verification.

### 3.1. TDC Core

**Figure 3:**
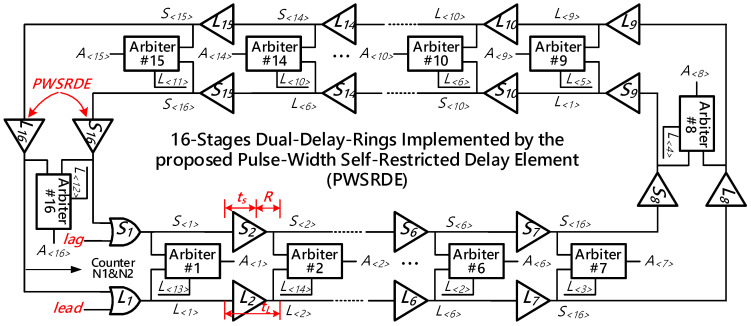
The architecture of the TDC core implemented by the 16-stages dual delay-rings.

In our design, the TDC core is mainly implemented by a pair of 16-stages delay-rings: the outer ring for the ‘*Lead*’ signal and the inner ring for the ‘*Lag*’ signal. As depicted in Figure 3, the delay-rings are formed by connecting the outputs of the last delay elements to the inputs of the first delay elements, and each delay-ring consists of 16 identical delay elements, which are all realized by the proposed PWSR delay element. Besides, a 2-input *OR* gate replaces a delay element as the first delay stage and is adopted to capture the original input signals. In the dual rings, the delay difference between the *OR* gates pair is exactly equal to that between the delay element pair. To be specific, the outer and inner rings are respectively constructed with *L*_1_~*L*_16_ and *S*_1_~*S*_16_. The delay time of the *L_i_*, namely *t_L_*, is set to be slightly larger than that of *S_i_*, namely *t_S_*. As the measurement begins, the ‘*Lead*’ signal first propagates through the outer delay-ring circularly and it generates a delayed output after each delay element. The ‘*Lag*’ signal arrives later but it experiences smaller delays in the delay-ring and chases the ‘*Lead*’ signal. Besides, the 16 arbiters (*A*_1_~*A*_16_) are connected to the outputs of *L*_1_~*L*_16_ and *S*_1_~*S*_16_ to monitor the states of the dual delay-rings. In principle, the time-resolution, namely *R*, is equal to the difference between the two delay elements, which can be calculated as:

(2) $$R=t_{L}-t_{S}.$$

Overall, the operation principle of converting a time-interval into digital codes employing the proposed TDC core can be concluded into three stages.

- During the 1st stage, merely the ‘*Lead*’ signal is fed into the 16-stages dual delay-rings. Before the ‘*Lag*’ signal enters the inner delay-ring, the effective pulse of the ‘*Lead*’ signal will be transmitted through the outer delay circularly. In this stage, the TDC core operates in its coarse measurement mode. Commonly, the 1st counter, which can be found in Figure 2, will increase every alternate cycle. Hence, the time-resolution in the 1st stage is 16*t_L_*. As the valid rising edge of the ‘*Lag*’ signal appears, the 1st counter will be halted immediately, and then its count value is stamped as *N*_1_. The value of the least significant bit (LSB) of *N*_1_ is 16*t_L_*.
- In the 2nd stage, the ‘*Lag*’ signal also arrives in the inner delay-ring. In this case, the ‘*Lead*’ signal and ‘*Lag*’ signal simultaneously propagate through the 16-stages dual delay-rings. The TDC core is switched into fine measurement mode. The residue of the time-interval will be quantized with a precision of *R = t_L_-t_S_*. The 2nd counter connected to the output terminal of delay element *L*_16_ will increase as the ‘*Lag*’ signal transmits a full circle. Once the ‘*Lag*’ signal catches up with the ‘*Lead*’ signal in the dual delay-rings, the 2nd counter will be halted immediately, and its count value is recoded as *N*_2_.
- During the 3rd stage, the 16 arbiters promptly compare the outputs of the 16-stages dual delay-rings to exactly locate the position where the first ‘*01*’ transition occurs. The outputs of the arbiters are combined to form a 16-bit thermometer codes. They are then converted to a 4-bit binary code *N*_3_ by the TM2B encoder for further processing. The measured time difference in this stage can be calculated as *N*_3_*(*t_L_−t_S_*).

**Figure 4:**
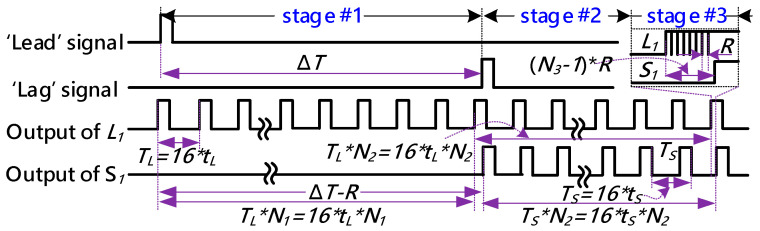
The timing diagram of the TDC core.

Figure 4 describes a simplified timing diagram of the TDC core. The time-interval between the ‘*Lead*’ signal and the ‘*Lag*’ signal, namely Δ*T*, can be expressed as:

(3) $$\Delta T=N_{1}\times 16t_{L}+N_{2}\times 16\left(t_{L}-t_{S}\right)+N_{3}\times \left(t_{L}-t_{S}\right).$$

In Equation (3), *N*_1_ and *N*_2_ represent the outputs produced by the first and second counter, respectively. *N*_3_ stands for the value of the thermometer codes, which indicates the location where the first ‘*01*’ transition occurs in the 16-stages arbiter ring. *t_L_* and *t_S_* are the delay values of the delay elements in the outer and inner rings, respectively. Regularly, *N*_1_, *N*_2_, and *N*_3_ are merged by the register banks to form the final output of the TDC.

As a matter of fact, the proposed TDC exhibits a wide dynamic range while keeping a relatively compact size. This benefits from the fact that it places the delay elements in a ring format so that reusing the delay elements becomes achievable. Moreover, the proposed TDC is competent to realize sub-gate time-resolution thanks to its vernier architecture based on the dual delay-rings. Hence, it is desirable in 3D imaging applications.

### 3.2. PWSR Delay Element

Delay element is particularly critical for a TDC as it vastly influences the TDCs’ performance, for example, the achievable time precision. To date, several kinds of delay elements been reported in the literature, including inverter, buffer, switched inverter, and so on. Normally, the inverter-based delay elements seem to be more attractive since their structure are compact and they can provide time-resolution of tens of picosecond. However, the delay time of such inverter-based delay elements is strongly influenced by the process variations and cannot be precisely controlled. In this paper, we proposed a novel PWSR delay element that is capable of providing accuracy and steady time delay to build the dual delay-rings.

**Figure 5:**
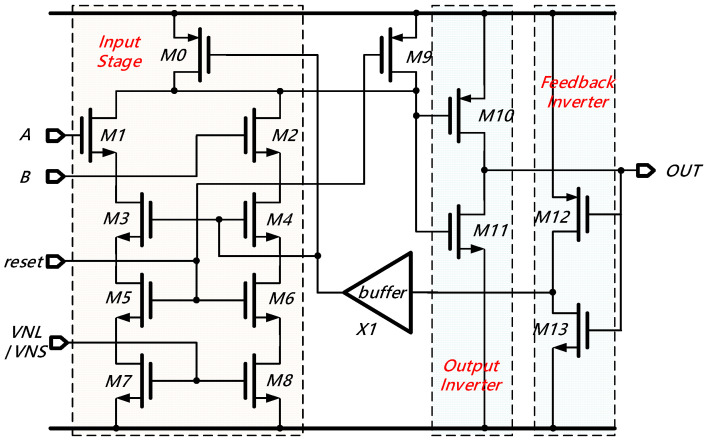
Schematic of the proposed pulse-width self-restricted (PWSR) delay element.

Figure 5 exhibits the schematic of the proposed PWSR delay element. Fundamentally, the PWSR delay element comprises four blocks, namely the input stage (*M0-M8*), the output inverter (*M10-M11*), the feedback inverter (*M12-M13*), and the buffer (*X1*). In the input stage, *M1*, *M3*, *M5*, and *M7* along with *M2*, *M4*, *M6*, and *M8* form two identical current branches to alleviate the influence of device mismatch. The input signals ‘*A*’ and ‘*B*’ are respectively connected to the gates of *M1* and *M2*. It should be noted that in each delay-ring, only the first delay element (*L*_1_ or *S*_1_) employs two sets of input ports simultaneously. The remaining delay elements merely employ one of the two input ports, and the other input port is directly connected to the ground. Moreover, the gates of transistors *M5*, *M6,* and *M9* are controlled by the ‘*rese*t’ signal. Circuit resetting is carried out by applying a negative-going step to the ‘*rese*t’ node before the initial state and after each quantization. In this case, the output of the PWSR delay element will be clamped at a low level. The transistors *M7* and *M8* act as a pair of current sinks, whose values are controlled by external voltage ‘*VNL*’ or ‘*VNS*’. Accordingly, the delay time can be effectively programmed by adjusting ‘*VNL*’ or ‘*VNS*’. The output stage is constituted with transistors *M10* and *M11* and it is utilized for enhancing the driving capability of the PWSR delay element during the transition operation. The feedback inverter is composed of transistors *M12* and *M13*, it sends the output signal back to the input stage through a unity-gain buffer. When a positive pulse is applied to the input terminal, a positive-going step will subsequently appear in the output terminal after a certain delay time controlled by ‘*VNL*’ or ‘*VNS*’. Once the output signal goes high, *M3* and *M4* will be cut off, and both branches of the current sinks will not consume any static power. Meanwhile, transistor *M0* will go into the saturation region. Consequently, the output of the PWSR delay element will be pulled down to the ground. Thanks to the fast feedback chain, the pulse-width of the output signal will be restricted within an ultra-narrow range and this makes the PWSR delay element be suitable for high precision TDC.

**Figure 6:**
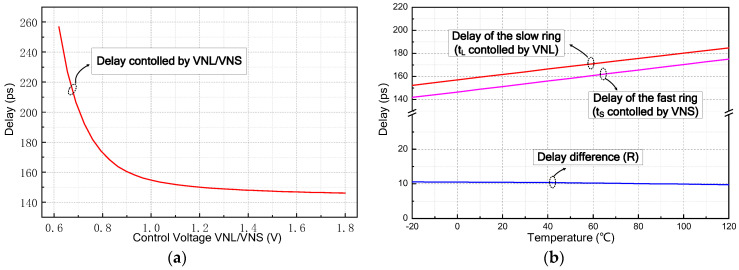
The simulated results of the proposed PWSR delay element: (**a**) dependence of the delay time on the controlled voltage *VNL*/*VNS* and (**b**) dependence of the delay time on temperature.

The performance of our proposed PWSR delay element is preliminarily verified by simulation. Figure 6a shows the dependence of the delay time on the external control voltage ‘*VNL*/*VNS*’. As the control voltage successively increases from 0.6 V to 1.8 V, the delay time decreases from 243 ps to 143 ps. In our design, the delay elements in the outer ring should keep a longer delay time than those in the inner ring, so ‘*VNL*’ is tuned to be smaller than ‘*VNS*’. In practice, the values of ‘*VNL*’ and ‘*VNS*’ should be determined carefully to acquire a preferable time-resolution. Figure 6b illustrates the dependence of the delay time on temperature. ‘*VNL*’ and ‘*VNS*’ are set to 0.9 V and 1.1 V, respectively. The simulated *t_L_* and *t_S_* are about 149 ps and 139 ps and thus a high time-precision of 10 ps can be successfully achieved. Significantly, the temperature coefficients of *t_L_* and *t_S_* almost equal to each other, which are 0.214 ps/°C and 0.221 ps/°C, respectively. This virtually makes the delay difference nearly immune to the temperature variation and enables the proposed PWSR delay element to produce a steady time-resolution.

**Figure 7:**
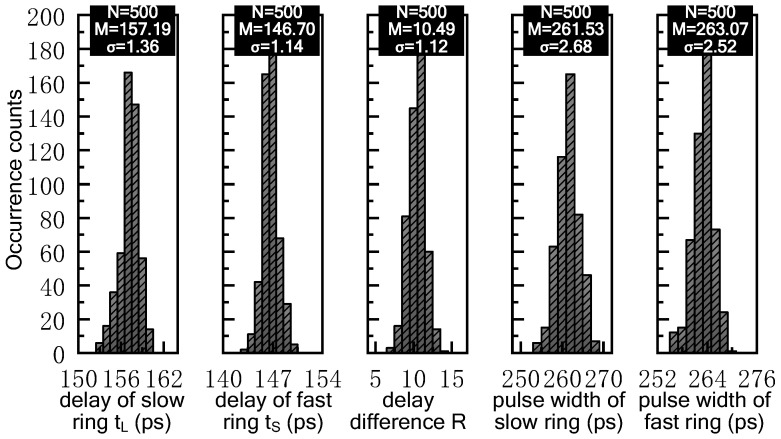
Monte Carlo simulation results of the proposed PWSR delay element.

Apart from the above single-point analysis, we also evaluate the performance of our proposed PWSR delay element by Monte Carlo simulation. The statistical analysis of five critical parameters have been performed, including the delays (*t_L_*, *t_S_*) and pulse-widths of the delay elements in the fast and slow rings as well as the time-resolution. ‘*VNL*’ and ‘*VNS*’ are set to 0.95 V and 1.70 V, respectively. As shown in Figure 7, for the delay element in the slow ring that is controlled by ‘*VNL*’, the mean value and the standard deviation of its propagation delay are 157.19 ps and 1.36 ps, respectively. Likewise, for the delay element in the fast ring that is controlled by ‘*VNS*’, the mean value and the standard deviation of its propagation delay are 146.7 ps and 1.14 ps, respectively. Besides, the standard deviation of time-resolution ‘*R*’ is merely 1.12 ps. Furthermore, it can be observed that the mean values and standard deviations of the pulse-widths for a PWSR delay pair are 261.53 ps, 2.68 ps, and 263.07 ps, 2.52 ps, respectively. This signifies that the proposed PWSR delay element can provide relatively stabilized delay and pulse-width, and thus it is suitable for high-resolution TDC.

### 3.3. Fully Symmetric Arbiter

In the TDC architecture being worked upon, the arbiters are responsible for monitoring the states of the dual delay-rings and locate the position where the ‘*Lag*’ signal catches up with the ‘*Lead*’ signal [25]. Optimization of the arbiters is important for designing high-performance TDC. Normally, DFF has been widely adopted as an arbiter in conventional TDCs owing to its superiorities of small area and low power. However, most DFF-based arbiters usually fail to deal with the errors stemming from the inconsistency between the input paths, which mainly originates from the PVT variations. In this paper, we designed a novel arbiter architecture based on a fully symmetric topology for the proposed TDC.

**Figure 8:**
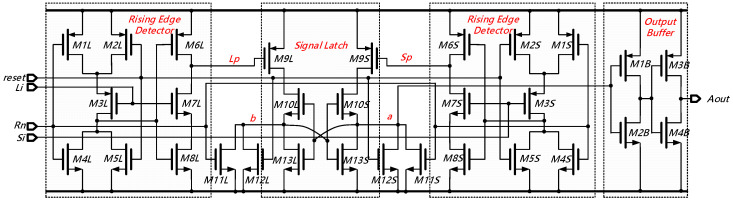
Schematic of the proposed fully symmetric arbiter.

The proposed fully symmetric arbiter is schematically illustrated in Figure 8. In short, it consists of a pair of identical rising-edge detectors (*M1L*~*M8L* and *M1S*~*M8S*), a signal latch (*M9L*~*M13L* and *M9S*~*M13S*), and an output buffer (*M1B*~*M4B*). As shown in Figure 8, not only the left and right rising-edge detectors are symmetrical to each other, but also the signal latch holds a fully symmetric topology. In the arbiter being worked upon, the input signals ‘*Li*’ and ‘*Si*’ are respectively fed into the left and right rising edge detection circuits. Both ‘*Rn*’ and ‘*rese*t’ are reset control signals: one for local resetting and the other for global resetting. Once a negative pulse is applied to the reset signals, the arbiter will be shut down since the rising edge detectors are disabled. Otherwise, the arbiter begins to compare the arriving sequence of ‘*L_i_*’ and ‘*S_i_*’, which respectively propagate through the outer and inner delay-rings in the TDC core. When a positive pulse arrives at its input terminal, each of the edge detectors will output a negative step. Meanwhile, the output of the edge detector will be temporally stored and subsequently transformed into a positive pulse by the signal latch. Finally, a positive-going pulse will be sent to the output terminal with a strong driving capability through the output buffer. Consequently, the arbiter outputs ‘*0*’ when the signal ‘*Li*’ reaches the input node firstly, and this means the ‘*lag*’ signal has not caught up to the ‘*lead*’ signal yet. Likewise, the arbiter outputs ‘*1*’ when signal ‘*Si*’ arrives earlier, in other words, the ‘*lag*’ signal catches up to the ‘*lead*’ signal. In this way, the location where the first ‘*01*’ transition occurs in the dual delay-rings can be efficiently detected and then used to latch the *2nd* counter.

**Figure 9:**
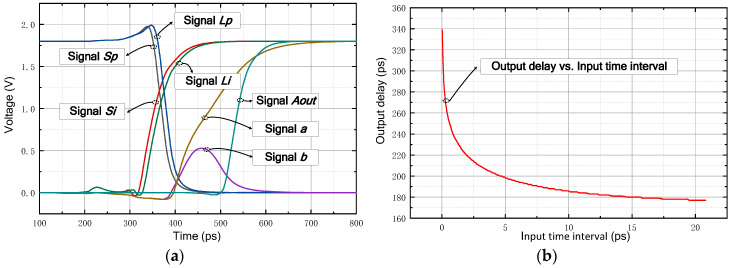
The simulated results of the proposed fully symmetric arbiter: (**a**) the transient result and (**b**) dependence of the output delay on the input time-interval.

The simulated transient result of the proposed fully symmetric arbiter is shown in Figure 9a. The output waveforms of several critical nodes are provided for analysis, including the input signals ‘*S_i_*’ and ‘*L_i_*’, the output signals ‘*A_out_*’, and the inner signals ‘*L_p_*’, ‘*S_p_*’, ‘*a*’ and ‘*b*’. The time-interval between ‘*S_i_*’ and ‘*L_i_*’ is set as 10 ps. As shown in Figure 9a, when ‘*Si*’ arrives earlier than ‘*Li*’, ‘*S_p_*’ and ‘*L_p_*’ are pulled up to high level successively. Meanwhile, the inner node ‘*a*’ outputs a higher voltage than node ‘*b*’; thereafter, signal ‘*a*’ is boosted to the supply voltage by the output buffer and finally sent to the output node ‘*A_out_*’. Besides, Figure 9b exhibits the correlation of the arbiter delay time and the input time-interval. It is apparent that the output delay increases dramatically when the time-interval shrinks to less than 1 ps. As the input time-interval excesses 10 ps, the arbiter delay is beginning to stabilize.

### 3.4. Compact TM2B Encoder

In the proposed TDC, the TM2B encoder is utilized to converting the output of the arbiter ring from a 16-bit thermometer code to a 5-bit binary code for further processing. Conventional TM2B encoder is usually complicated and occupies a relatively large die area. However, this is exceedingly undesirable for the TDC in a 3D image sensor. To address this issue, we proposed a compact TM2B by elaborately exploiting the signals’ special characteristics.

**Figure 10:**
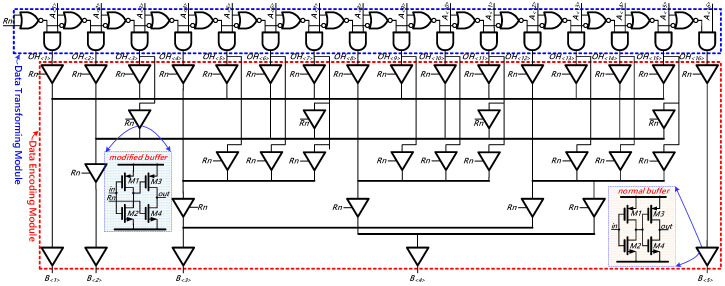
The architecture of the thermometer-to-binary encoder.

**Figure 11:**
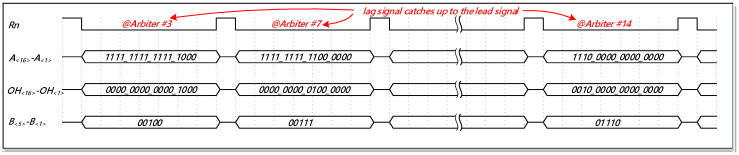
The timing diagram of the proposed thermometer-to-binary (TM2B) encoder.

The architecture of our proposed TM2B encoder is shown in Figure 10. It can be generally divided into two main components: namely the data transforming module and the data encoding module. *A_<_*_16*>*_~*A_<_*_1*>*_ are the outputs of the 16-stages arbiter chain. *R_n_* is a local reset signal. The operation principle of the proposed TM2B encoder can be also divided into two basic phases. In the first phase, the data transforming module converts the thermometer code (*A_<_*_16*>*_~*A_<_*_1*>*_) into a 16-bit one-hot code (*OH_<_*_16*>*_~*OH_<_*_1*>*_), in which all of the elements are ‘0’ except for one, which has ‘1’ as its value. Ideally, ‘*1*’ represents the initial arbiter whose output first changes from ‘*0*’ to ‘*1*’ and specifies the location where the ‘*Lag*’ signal catches up with the ‘*Lead*’ signal. In the second phase, the data encoding module converts the 16-bit one-hot code (*OH_<_*_16*>*_~*OH_<_*_1*>*_) into a 5-bit binary code, which will be merged with the outputs of the aforementioned counters. To make it explicit to follow, Figure 11 analyses the timing diagram of our proposed TM2B encoder.

In the proposed TM2B encoder, the data transforming module is built with 16 sets of cascaded transforming elements formed by logic gates. The data encoding module is mainly implemented by two kinds of buffers, namely modified buffer and normal buffer. As shown in Figure 10, the normal buffer is made up of a pair of inverters connected in series; whereas the modified buffer is realized by interrupting the connection between the gate of *M4* and the output of the first inverter. In addition, a local reset signal ‘*Rn*’ is also inserted in the modified buffer. As discussed before, the arbiters will be reset as a negative-going pulse is applied to ‘*Rn*’ and thus the input signal (*A_<_*_16*>*_~*A_<_*_1*>*_) of the TM2B encoder will remain zeros. Meanwhile, the gate of transistor *M3* in the modified buffer will be pulled up to ‘*1*’ so that *M3* will be cut off. Furthermore, since there is merely one ‘*1*’ in the one-hot code (*OH_<_*_16*>*_~*OH_<_*_1*>*_), the modified buffers in our proposed TM2B encoder can operate properly even though some of their output terminals are directly shorted. Based on the analysis, the outputs of the proposed TM2B encoder can be expressed as follows:

(4) $$B_{\langle 1\rangle }=A_{\langle 1\rangle }+A_{\langle 3\rangle }+A_{\langle 5\rangle }+A_{\langle 7\rangle }+A_{\langle 9\rangle }+A_{\langle 11\rangle }+A_{\langle 13\rangle }+A_{\langle 15\rangle }.$$

(5) $$B_{\langle 2\rangle }=A_{\langle 2\rangle }+A_{\langle 3\rangle }+A_{\langle 6\rangle }+A_{\langle 7\rangle }+A_{\langle 10\rangle }+A_{\langle 11\rangle }+A_{\langle 14\rangle }+A_{\langle 15\rangle }.$$

(6) $$B_{\langle 3\rangle }=A_{\langle 4\rangle }+A_{\langle 5\rangle }+A_{\langle 6\rangle }+A_{\langle 7\rangle }+A_{\langle 12\rangle }+A_{\langle 13\rangle }+A_{\langle 14\rangle }+A_{\langle 15\rangle }.$$

(7) $$B_{\langle 4\rangle }=A_{\langle 8\rangle }+A_{\langle 9\rangle }+A_{\langle 10\rangle }+A_{\langle 11\rangle }+A_{\langle 12\rangle }+A_{\langle 13\rangle }+A_{\langle 14\rangle }+A_{\langle 15\rangle }.$$

(8) $$B_{\langle 5\rangle }=A_{\langle 16\rangle }.$$

## 4. Experimental Results and Discussions

**Figure 12:**
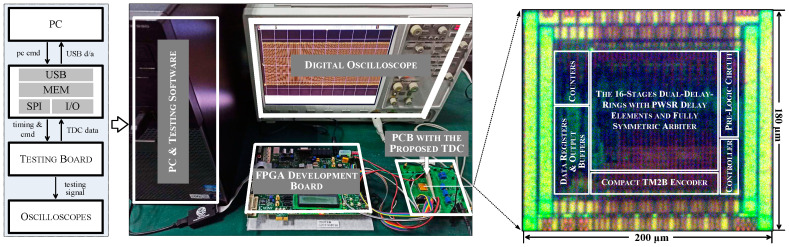
The micrograph of the proposed TDC (**right**) and the chip test system (**left**).

The proposed vernier TDC, which employing a 16-stages dual delay-rings based on PWSR delay element and fully symmetric arbiter, has been implemented and fabricated by standard 0.18-µm one-poly four-metal (1P4M) CMOS process. The die micrograph of the prototype is shown in the right half of Figure 12. The active area of the proposed circuit is about 200 µm × 180 µm. The left half of Figure 12 exhibits the setup of the chip test system. The Xilinx Virtex-5 FPGA development board serves as a microcontroller. It not only transmits the control signals to the testing board through SPI and receives valid data from TDC chip, but also sends the acquired data to the PC and receives the configuration instructions through a USB port in the meantime. The Keysight Technologies digital oscilloscope is employed for ripple measurement. Besides, the PC behaves like a host. It programs the configuration files to the FPGA device and manages the collected datasets. The PCB board contains the fabricated TDC and several auxiliary circuitries that enable the proposed TDC to work properly.

Ideally, the propagation delay of each delay element can be obtained by directly measuring the periods of signal ‘*L*_16_’ and signal ‘*S*_16_’, which represent the outputs of the last delay elements in the outer and inner delay-rings, respectively. However, this is usually impractical because both ‘*L*_16_’ and ‘*S*_16_’ have an extremely narrow pulse-width (within 200 ps) and they may vanish immediately once they are directly connected to a pad with large capacitance. To address this issue, we have elicited the internal signals, ‘*L*_16_’ and ‘*S*_16_’, to the external pad through a DFF and a series of buffers. The data input node is connected to ‘*1*’ and its reset is connected to ‘*L*_8_’ or ‘*S*_8_’. ‘*L*_16_’ or ‘*S*_16_’ serves as a clock for the DFF. In this way, the pulse-widths of ‘*L*_16_’ and ‘*S*_16_’ are effectively extended to 50% of the signal period. This makes the periods of signal ‘*L*_16_’ and signal ‘*S*_16_’ measurable.

**Figure 13:**
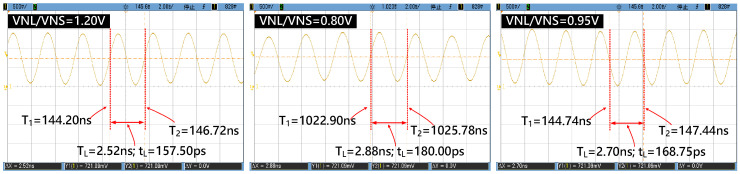
Measured output waveforms of the pulse-width widened ‘L16/S16’ when ‘VNL/VNS’ are set as 1.20 V, 0.80 V, and 0.95 V.

Figure 13 shows the measured output waveforms of the pulse-width widened ‘*L*_16_/*S*_16_’ when the delay time control voltages ‘*VNL*/*VNS*’ are respectively set as 1.20 V, 0.90 V, and 0.95 V. As can be seen from Figure 13, the output waveform has become a sine wave waveform, rather than a normal pulse waveform obtained in the simulated results. This kind of distortion mainly arises from the large load capacitance introduced by the oscilloscope’s probe. As a matter of fact, it hardly changes the signal period, so the measured waveform is still meaningful for chip verification. As shown in Figure 13, the periods of ‘*L*_16_/*S*_16_’, namely ‘*T_L_*’, are respectively measured as 2.52 ns, 2.88 ns, and 2.70 ns as the control voltages ‘*VNL*/*VNS*’ are successively set as 1.20 V, 0.90 V, and 0.95 V. Furthermore, by dividing the measured period by the number of stages (16), the delay time of each delay element can be obtained as 157.50 ps, 180.00 ps, and 168.75 ps. From the graph above we can see that there is a significant negative correlation between the delay time of each delay element and the control voltage. Figure 13 demonstrates the functional correctness of our proposed circuit.

**Figure 14:**
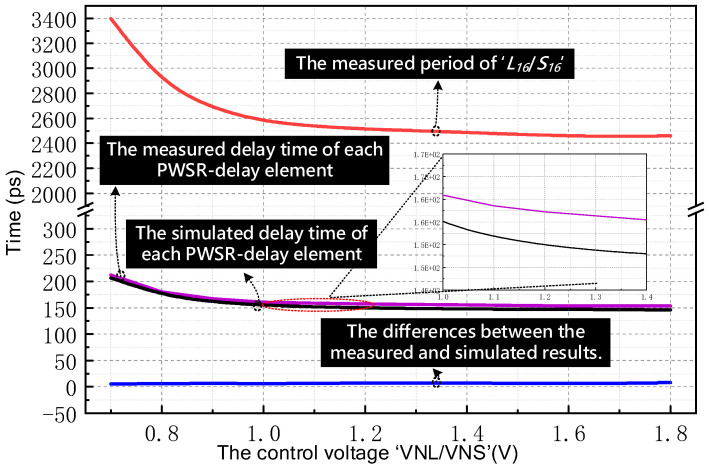
Measured and simulated results at different control voltage.

Figure 14 compares the measured and simulated results with successive increases in control voltages, and the inset of Figure 14 shows a magnified view of a portion of the results. In the beginning, the measured period of the 16-stages delay-ring ‘*L16*/*S16*’ keeps decreasing as the control voltage varies from 0.8 V to 1.8 V. Then, it tends to be stable as the control voltage exceeds 1.2 V. Obviously, this tendency is consistent with the former theoretical analysis. Besides, the measured and simulated results in terms of the delay time of each PWSR-delay element are also provided in Figure 14. It can be seen that no significant difference between the measured delay curve and the simulated delay curve is evident. More specifically, the calculated delay difference between the measured and simulated results swings from 5.9 ps to 9.6 ps. This demonstrates that the both proposed PWSR-delay element as well as the dual delay-rings in the fabricated TDC can work properly as expected.

In our proposed TDC, if the control voltages of the delay elements in the fast and slow rings, namely ‘*VNL*’ and ‘*VNS*’, are respectively set as 0.80 V and 0.95 V. The measured delay time of ‘*Li*’ and ‘*Si*’, namely ‘*t_L_*’ and ‘*t_S_*’, can be respectively measured as 180.00 ps and 168.75 ps. Hence, the realistic distinguishable time precision, namely ‘*R*’, can be calculated as 11.25 ps. According to Equation (3), the TDC output is formed by three parts: the high-order 5-bits *N*_1_, the middle-order 4-bits *N*_2_, and the low-order 4-bits *N*_3_. The values of LSBs for these three parts can be respectively calculated as 2.88 ns, 180 ps, and 11.25 ps, and thus the maximum time-interval that our proposed TDC can deal with is 92.1 ns. When the result is applied to a 3D image sensor with an ideal SPAD device, it corresponds to a detectable range of 13.82 m and a resolution of 1.7 mm. This indicates the ultimate performance of a 3D imaging system using our proposed TDC. Furthermore, the proposed TDC can also satisfy the demand for long-distance detection applications by simply adjusting the delay difference of the PWSR delay elements in the dual delay-rings. This flexibility along with its superior characteristics of wide dynamic range and high precision makes the proposed TDC well suited for 3D imaging applications.

To verify the functionality of the fabricated TDC preliminarily, we have fed 10 discontinuous time-intervals to its input terminals and then measured the corresponding digital codes at the output nodes. The measured results have been summarized in Table 1. It is apparent from this table that the measured TDC output can correctly follow the input time-intervals within a reasonable time error.

**Figure 15:**
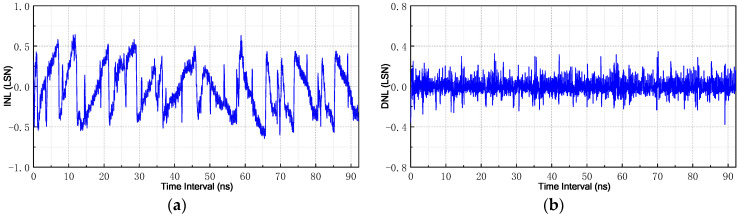
Measured nonlinearities of the proposed TDC: (**a**) integral nonlinearity (INL) and (**b**) differential nonlinearity (DNL).

Furthermore, TDC nonlinearities are also evaluated as they impact negatively upon the system resolution. In the literature, the most widely-used metrics to characterize TDC nonlinearities are differential nonlinearity (DNL) and integral nonlinearity (INL) [26]. DNL refers to the difference between each actual output step-width and the ideal output step-width. It provides a mechanism to describe the effects of environmental noise in a simple and uniform way. For an ideal TDC, the output steps have a uniform width. Meanwhile, INL can be defined as maximum deviation of the actual input-output characteristic from the ideal transfer characteristic. INL can be estimated using DNL at each step by calculating the cumulative sum of DNL errors up to that point. In this work, we have obtained the INL and DNL using the code density method and exhibited the results in Figure 15. The worst measured INL and DNL are respectively 0.65 LSB and 0.38 LSB, and both of them are less than 1 LSB. The nonlinearities are normally introduced by a variety of non-ideal effects (such as IR-drop, PVT variations, mismatch, etc.), and they can be partially compensated by circuit optimization [27].

**Figure 16:**
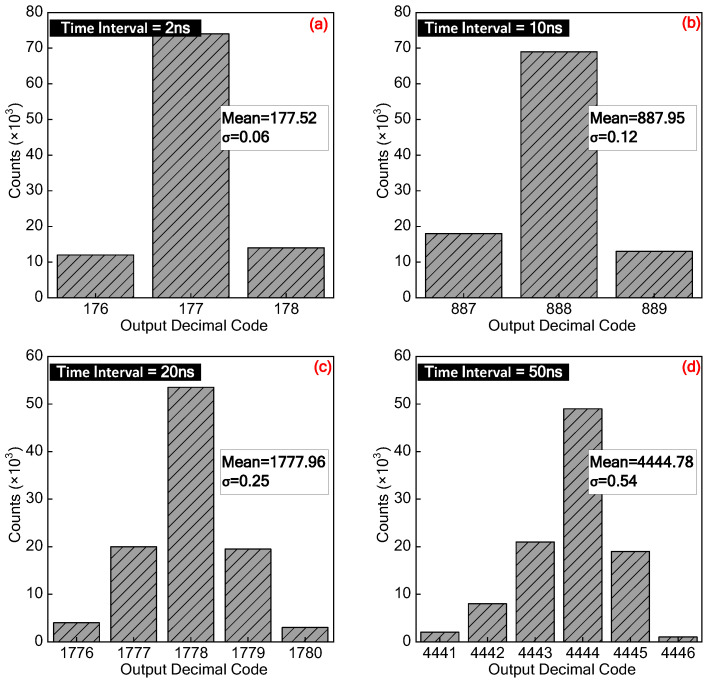
Measured TDC output code distributions as the constant time-intervals are (**a**) 2 ns, (**b**) 10 ns, (**c**) 20 ns, and (**d**) 50 ns.

Additionally, the precision of our proposed TDC has also been evaluated by carrying out single-shot measurement. We have collected and analyzed the TDC’s output codes in almost 100,000 consecutive measurements with a fixed input time-interval. Figure 16 presents the statistic histogram of the measured output codes for 4 cases of different fixed input time-interval. From this Figure, we can see that the measured mean values of the TDC output codes for inputs of 2 ns, 10 ns, 20 ns, and 50 ns are respectively 177.52, 887.95, 1777.96, and 4444.78, which sequentially correspond to time-intervals of 1.997 ns, 9.989 ns, 20.002 ns, and 50.004 ns. Moreover, the histogram output codes spread across several positions approximating the Gaussian error distribution, and all of the achieved standard deviations for these 4 cases stay below 1 LSB.

Table 2 summarizes the measured performance of the porotype TDC and compares it with the previous state-of-the-art TDCs. The results show that the entire TDC chip consumes 1.6 mW from a 1.8 V power supply with an operation frequency of 15 million samples per second (MSPS), which is comparable to other works. Besides, it can be seen from Table 2 that the proposed TDC in this work reaches a large dynamic range while keeping a relatively small time-resolution. Despite the TDC in [22] keeps a wider dynamic range than this work, its minimum resolvable is poor, which is about 6 ps larger than that of our proposed TDC. Furthermore, despite having been fabricated by a former generation process with a large minimum feature size, the proposed TDC still occupies a smaller die area than most of the previous works. The superior performances of dynamic range, high time-resolution, and compact die size primarily arise from the inherent characteristics of vernier-based dual delay-rings and PWSR delay elements. Additionally, since both the proposed arbiter and PWSR delay element employ a fully symmetric topology, the proposed TDC exhibits comparable linearity performance in comparison to other state-of-the-art works. In summary, the experimental results demonstrate that the proposed TDC in this work is capable of achieving high dynamic range and resolution while maintaining other parameters (such as power consumption, linearity, die size, etc.) within a reasonable range. Therefore, the proposed TDC is suitable for SPAD-based 3D imaging applications.

## 5. Further Research: A 3D Image Sensor Based on the Proposed TDC

As mentioned in Section 1, the 3D sensor has been grabbing enormous interest from researchers due to its outstanding characteristics. Generally, D-ToF based 3D image sensor is mainly comprised of two essential modules, respectively the SPAD array and the TDC [32]. The specific objective of this paper is to address the design challenges of the TDC. Other related works, such as SPAD design and sensor chip implementation, are also undertaken and they will be thoroughly discussed in other papers. Here, we would like to briefly introduce our ongoing work: a 3D image sensor based on the proposed TDC.

**Figure 17:**
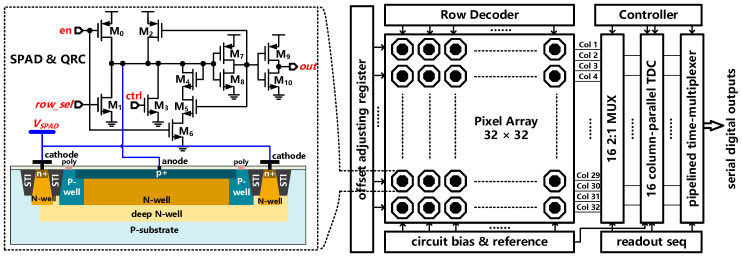
Block diagram of the 3D image sensor based on our proposed TDC (**right**) and its pixel circuit schematic (**left**).

The overall architecture of the 3D image sensor based on our proposed TDC is illustrated in Figure 17. As shown in the right part of Figure 17, the proposed 3D image sensor consists of a pixel array, a row decoder and selector, an offset adjusting register, a bias and reference circuit, a readout multiplexer array, a bank of 16 column-parallel TDCs, a pipelined time-multiplexer, and some other ancillary circuitry. The pixel circuit of this 3D image sensor is schematically shown in the left part of Figure 17. The proposed pixel mainly contains a light-sensitive unit of SPAD and an in-pixel quenching-and-reset circuit (QRC). The SPAD is realized with a p-n junction formed by p+ layer diffused on a deep n-well. A p-well loop is used as the guard ring. The central point of p+ diffusion and the edge of deep n-well are contacted out through the metal layer as the anode contact and cathode contact, respectively. The in-pixel QRC is in charge of not only quenching the large avalanche current but also making sure the SPADs back to bias above the breakdown voltage for the next avalanche event. Overall, the 3D image sensor totally contains 1024 pixels which are placed in 32 rows and 32 columns. To reusing the TDCs, 16 2-to-1 column-multiplexers are placed between the pixel array and the TDC array. A bank of 16 column-parallel TDCs is used to simultaneously process half row SPADs in each measurement. Each TDC channels fit within the pitch of two pixel-column. A full scan of the whole SPAD array takes 64 measurement cycles. The row selection for the connection to TDCs is performed before every measurement based on control data. Moreover, the pipelined time-multiplexer located in the column bus is used to select the pixel, then converts its parallel data into serial bits and sends them out of the chip serially.

At present, the electrical design and simulation of the proposed 3D image sensor have been almost completed, and the physical design and verification are being carried out. Once the chip is fabricated and tested, we will write another paper, whose content focuses on system implementation, to report the ongoing research.

## 6. Conclusions

The 3D imaging system based on a SPAD image sensor has been grabbing enormous interest from researchers due to its outstanding characteristics. In a typical SPAD image sensor, a TDC behaves like a digital stopwatch to precisely quantize the time-interval into a digital code. Generally, the design challenges of the TDC for a SPAD image sensor include but are not limited to wide dynamic range, small die area, high time-resolution, and low power consumption. In this paper, a 13-bit, 12-ps resolution vernier TDC based on dual delay-rings for a SPAD image sensor has been fully described, designed, implemented, and evaluated. Specifically, a PWSR delay element is proposed to realize a precise resolution. Its pulse-width is successfully restricted within an ultra-narrow range by a fast feedback chain. Besides this, its delay can be effectively programmed by adjusting the control voltage. Moreover, a compact and fast arbiter based on fully symmetric topology is presented and it approximately immunes to the PVT variations. Furthermore, a prototype vernier TDC circuit based on a 16-stages dual delay-rings constructed with the proposed PWSR delay element and fully symmetric arbiter is designed and implemented using standard 0.18-µm CMOS technology. The total active area of the proposed circuit is about 0.04 mm^2^ and the power consumption is nearly 1.6 mW under a 1.8 V power supply with an operating frequency of 15 MSPS. The proposed TDC circuit achieves a 13-bit dynamic range and an 11.25 ps resolution. This means the maximum time-interval that our proposed TDC can deal with reaches 92.1 ns. The worst measured INL and DNL are respectively 0.65 LSB and 0.38 LSB. Hence, the proposed TDC can find a useful application in 3D imaging applications.

## Figures and Tables

**Table 1:** Measured time-to-digital converter (TDC) outputs at 10 discontinuous input time-intervals.

Input Time-Interval	Binary Outputs	Decimal Outputs	Measured Time-Interval	Error
2 ns	00000 1011 0010	178	2002.50 ps	+2.25 ps
4 ns	00001 0110 0011	355	3993.75 ps	−6.25 ps
10 ns	00011 0111 1001	889	10001.25 ps	+1.25 ps
20 ns	00110 1111 0010	1778	20002.50 ps	+2.50 ps
30 ns	01010 0110 1010	2666	29992.50 ps	−7.50 ps
40 ns	01101 1110 0011	3555	39993.75 ps	−6.25 ps
50 ns	10001 0101 1101	4445	50006.25 ps	+6.25 ps
60 ns	10100 1101 0110	5334	60007.50 ps	+7.50 ps
70 ns	11000 0100 1110	6222	69997.50 ps	−2.50 ps
80 ns	11011 1100 0111	7111	79987.50 ps	−1.25 ps

**Table 2:** Performance summary and comparisons.

Parameter	[21]	[22]	[23]	[28]	[29]	[30]	[31]	This Work
Technology (nm)	130	350	45	65	130	45	180	180
Power supply (V)	1.5	3.3	1.0	1.2	1.2	1.0	1.2	1.8
Frequency (MSPS)	15	100	215	50	2.4	1250	30	25
Time-resolution (ps)	8.0	17.2	9.4	4.8	7.3	25	5.3	11.25
Dynamic Range (bit)	12	15	9	7	11	5	8	13
Integral nonlinearity (INL) (LSB)	N/A	1.1	1.1	3.3	1.2	N/A	2.8	0.65
Differential nonlinearity (DNL) (LSB)	N/A	<0.9%	0.57	<1	3.2	0.25	0.9	0.38
Area (mm^2^)	0.05	0.30	0.08	0.07	0.03	0.36	0.05	0.04
Power (mW)	7.50	15.0	24.2	1.70	1.20	16.0	1.1	1.6

## Data Availability

Data sharing not applicable.
